# Practical estimation of cloud storage costs for clinical genomic data

**DOI:** 10.1016/j.plabm.2020.e00168

**Published:** 2020-05-15

**Authors:** Niklas Krumm, Noah Hoffman

**Affiliations:** Department of Laboratory Medicine, University of Washington, Seattle, WA, USA

## Abstract

**Background:**

Laboratories performing clinical high-throughput sequencing for oncology and germline testing are increasingly migrating their data storage to cloud-based solutions. Cloud-based storage has several advantages, such as low per-GB prices, scalability, and minimal fixed costs; however, while these solutions tout ostensibly simple usage-based pricing plans, practical cost analysis of cloud storage for NGS data storage is not straightforward.

**Methods:**

We developed an easy-to-use tool designed specifically for cost and usage estimation for laboratories performing clinical NGS testing (https://ngscosts.info). Our tool enables quick exploration of dozens of storage options across three major cloud providers, and provides complex cost and usage forecasts over 1–20 year timeframes. Parameters include current test volumes, growth rate, data compression, data retention policies, and case re-access rates. Outputs include an easy-to-visualize chart of total data stored, yearly and lifetime costs, and a “cost per test” estimate.

**Results:**

Two factors were found to markedly decrease the average cost per test: 1) reducing total file size, including through the use of compression, 2) rapid transfer to “cold” or archival storage. In contrast, re-access of data from archival storage tiers was not found to dramatically increase the cost of storage per test.

**Conclusions:**

Steady declines in cloud storage pricing, as well as new options for storage and retrieval, make storing clinical NGS data on the cloud economical and friendly to laboratory workflows. Our web-based tool makes it possible to explore and compare cloud storage solutions and provide forecasts specifically for clinical NGS laboratories.

## 1 Introduction

Clinical laboratories are increasingly evaluating and adopting cloud storage solutions for long term storage and archival of clinical high-throughput (“next-generation”) sequencing data. Migration from local (i.e., on-premise) storage to cloud-based storage is driven by a combination of breakneck growth of clinical sequencing data as well as dramatically decreasing costs for cloud storage itself. For example, in 2019, the cost to store one gigabyte is approximately seven-fold lower than Amazon Web Services’ (AWS) initial offering of the Simple Storage Service (S3) in 2006; moreover, the most economical storage option today (AWS Deep Glacier) is over 150-fold lower (Table 1 & Fig. 2).

**Table 1:** 

Vendor	Storage Tier (see legend for abbreviations)	Cost per GB-Month (a)	Retrieval Time	Retrieval Cost per GB (b)	Cost per Test (6 ​GB Exome over 10 years)		
					Strategy A	Strategy B	Strategy C
AWS	S3	2.1–2.3 cents	Immediate	–	$12.39	$3.29 (2 years S3 then 8 years Deep Glacier)	$0.88 (3 months S3 then 10 years Deep Glacier)
	S3-IA	1.25 cents	Immediate	1.0 cents	$6.77		
	Glacier	0.4 cents	3–5 ​h (typical); 1–5 ​min (expedited)	0.25–3.0 cents	$2.17		
	Deep Glacier	0.099 cents	12–48 ​h	0.25–2.0 cents	$0.54		
GCP	Regional	2.0–2.3 cents	Immediate	–	$10.83	$5.57 (2 years Regional then 8 years Coldline)	$4.09 (3 months Regional then 10 years Coldline)
	Nearline	1.0 cents	Immediate	1.0 cents	$5.41		
	Coldline	0.7 cents	Immediate	2.0 cents	$3.79		
	Archive (c)	0.25 cents	Immediate	5.0 cents	$1.35		
Azure	ZRS Hot	2.12–2.3 cents	Immediate	–	$12.40	$3.30 (2 years LRS Hot then 8 years LRS Archive)	$0.86 (3 months LRS Hot then 10 years LRS Archive)
	ZRS Cool	1.25 cents	Immediate	–	$6.77		
	LRS Hot	1.7–2.08 cents	Immediate	–	$9.92		
	LRS Cool	1.0–1.5 cents	Immediate	1.0 cents	$5.41		
	LRS Archive	0.099–0.2 cents	<15 ​h	2.0 cents	$0.54		

Strategy A: 1000 exomes per year (6TB generated per year), stored for 10 ​years ​at indicated storage level.

Strategy B: 1000 exomes per year (6TB generated per year), stored for 2 years in “hot” storage and 8 years in “cold” storage.

Strategy C: 1000 exomes per year (6TB generated per year), stored for 3 months in “hot” storage and 10 years in “cold” storage.

(Simulating 20 years total, no re-access).

All prices, features and storage classes are current as of May 2020. An updated, online version of this table can be found at https://ngscosts.info.

Not all storage classes, features and costs from each vendor are represented. Displayed classes are meant to be representative of range available from each vendor.

Prices are subject to change.

Abbreviations; AWS: Amazon Web Serivces, GCP: Google Cloud Platform, S3: Simple Storage Service, S3-IA: S3 Infrequent Access, ZRS: Zone-redundant storage, LRS: Locally-redundant storage.

Notes: (a) Ranges indicate when prices may vary by region and/or total data stored(b) Data retrieved to locations outside of the vendors cloud or cross-region may incur network transfer costs.(c) GCP Archive does not come with a Service Level Agreement for availability.

**Fig. 1:**
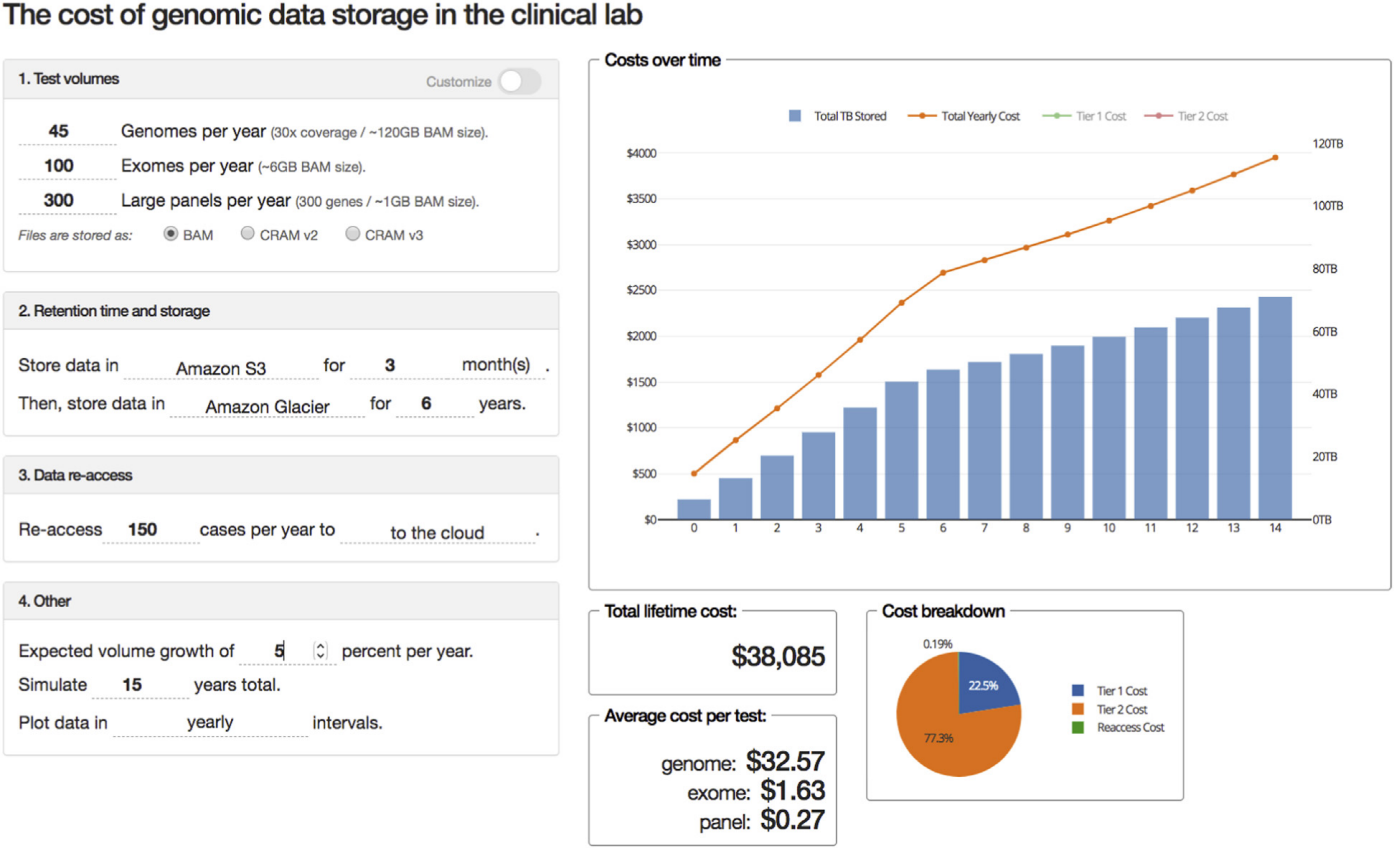
Screenshot from the application. Panels on the left side of the application enable control of parameters and customization. File sizes, test volumes, compression, storage tiers and retention times are configurable (see Methods for additional description of options). Outputs are interactively updated, and track total stored data over time, per-test marginal costs as well as the total lifetime cost.

**Fig. 2:**
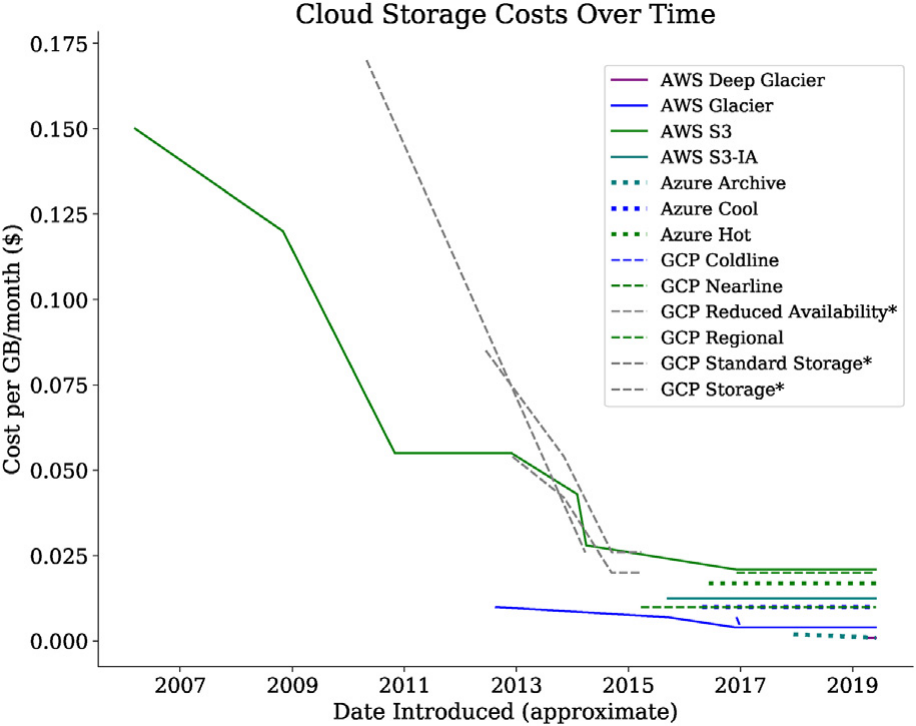
Historical prices of cloud storage across major cloud vendors and products over time. Storage tiers with an asterisk are either retired names or products. Please note: chart is based on historical or archived web content and dates of price drops or product introductions are approximate. AWS: Amazon Web Services, S3: Simple Storage Service, S3-IA: S3 Infrequent Access, GCP: Google Cloud Platform, GB: Gigabyte.

However, despite ostensibly simple usage-based pricing plans (marketed by most vendors as “pay for what you use”), it is surprisingly difficult to compare prices and provide forecasts of costs for long-term storage of clinical genomics data in the cloud. While all major cloud vendors provide online calculators for calculating costs [[1], [2], [3]], these calculators have significant shortfalls in the context of a clinical laboratory: 1) they do not estimate the marginal “cost per test” or a total lifetime cost, 2) they do not provide forecasts of costs when storage requirements may increase over time, and 3) they do not support estimation of costs when different storage tiers are desired for long term archiving.

To address these needs, we have developed an easy-to-use online calculator designed specifically for cost analysis and storage requirements estimation for laboratories performing clinical high-throughput sequencing. The tool as well as its source code is available at https://ngscosts.info. Here we discuss the use and assumptions of this tool, as well as describe various strategies and practical implementation details relevant to genomic data storage in the cloud.

## 2 Background

Amazon Web Services introduced their Simple Storage Service (S3) in 2006, at a price of $0.15 per Gigabyte-month (GB-month, Fig. 2) [4]. Since then, additional tiers of cloud storage have been introduced by both AWS as well as Alphabet’s Google Compute Platform (GCP) and Microsoft’s Azure Cloud. Astoundingly, the total cloud storage capacity today is estimated at more than 1 Zetabyte (or 10^21^ bytes) [5], while per GB-month costs have consistently fallen, with storage offerings now starting at less than $0.001 per GB-month.

For clinical sequencing laboratories, cloud storage promises to provide a solution for rapidly expanding storage requirements without expensive investment into local storage solutions. While exhaustive discussion of the implementation of cloud storage for clinical laboratories is outside the scope of this article (but see review by Carter [6]), several factors driving adoption of cloud-based storage solutions within the clinical lab are highlighted here:

### 2.1 Regulatory changes

CAP accredited laboratories must now address a new College of American Pathologists (CAP) checklist item (MOL.35870) which mandates a minimum retention time for read-level files (e.g., those with extensions of.fastq or.bam) of two years. Laboratories may opt to keep sequencing data for longer periods of time, especially for exome or whole-genome sequences obtained in the pediatric or neonatal setting.

Additionally, hospitals and academic institutions are increasingly aware of the opportunities afforded by cloud-based computing and storage services. Therefore, the implementation of Business Associate Agreements (BAAs) between clinical laboratories (or their host institutions) and cloud providers is becoming more routine, and cloud vendors are able to provide specific guidance around the implementation of services compatible with the Health Insurance Portability and Accountability Act (HIPAA) of 1996.

### 2.2 Durability and availability

A core benefit of cloud-based services is a high level of data durability and availability. *Durability* measures how resilient data is to long-term loss or corruption (e.g., due to natural disasters or physical loss of resources, technical errors or failures). Major cloud providers guarantee 99.999999999% durability; achieving this level of data protection requires multiple levels of geographic, physical and virtual redundancy, measures which are inaccessible to individual laboratories or even local institutional data centers. A related metric, data *availability*, measures how much downtime the cloud provider expects, independent of the data’s integrity. Data availability rates are typically around 99.99% or better (<1 ​h per year, although this represents an average and individual files or services may be impacted to greater extent).

### 2.3 New technical features

Cloud storage solutions have grown from being simple storage-only solutions to providing a complete ecosystem that can fully manage data, backups and auditing of data. Lifecycle policies can enable automatic migration of data from “hot” storage (i.e., immediately accessible at a higher cost) to “cold” or archival storage (i.e., lower per-GB cost achieved through re-access costs and delayed retrieval). In addition, configuration options have been added that enable encryption, auditing, access control for data containing Protected Health Inofrmation (PHI) under the HIPAA law, with no or minimal additional costs or complexity. Notably, many of these available technical options are significantly more challenging to implement for on-premise or local storage.

## 3 Methods

The application is a Python 3-based web application based on the Dash and Flask frameworks (Fig. 1), which supports real-time user interaction and rapid iteration and comparison of input settings. The application and source code is available at https://ngstools.info or https://github.com/nkrumm/storagecosts. User inputs include yearly test volumes for whole genomes, exomes and/or panels, with either canonical data storage size (120GB for genomes, 6GB for exomes or 1GB for panel tests), or customizable storage requirements for each test type. Lossy read compression techniques such as the.cram [7] format can be selected. Up to two storage tiers (or products) from cloud vendors may be specified. Retention time for two sequential tiers of storage can be specified as well; for the first tier, retention time can be defined in months. The total number of cases re-access per year is specified next, along with additional options such as total yearly growth in test volumes.

The cumulative amount of stored data, as well as associated costs are calculated iteratively with monthly compounding. Three principal types of costs are calculated: the per-month storage costs (which may be discounted with the increasing amounts stored), costs associated with transfer of data from storage to other cloud infrastructure or the internet (e.g., to local infrastructure), as well as any additional costs associated with re-accessing data from archival tiers. Both total volume of data stored, and total yearly (or monthly) costs are displayed in the main graphical output area. Additional outputs include the average per-test cost for each type of test specified, the total lifetime cost of storage, as well as a breakdown of costs by storage tier and re-access costs.

All cloud storage costs are parameterized in order to facilitate ongoing maintenance of the application when per-GB/month or other costs change, and if new storage tiers or additional cloud vendors are introduced.

## 4 Results

Table 1 describes the cost per GB-month for 12 different products from three major cloud vendors. For archival tiers, retrieval speed as well as cost are also listed. For some products, costs are listed as ranges, as they depend on the total amount of data stored, the geographic region selected for storage, and retrieval speed selected. Prices were current as of April 2020.

We find that recent price drops in cloud storage and the introduction of “deep archive” tiers of storage have dropped the cost of storing individual data files from panel-based or exome experiments to just pennies per year. For example, yearly storage costs for a 6GB file stored in AWS Deep Glacier or Azure Archive is an astounding $0.07 *per year*, or just $0.71 over ten years. On account of their larger size, we estimate the cost to store a single genome (120GB in size) to be approximately $14 over ten years. Taken in the context of reagent, laboratory overhead, staffing and other costs, the marginal cost to store sequencing data in archival storage tiers is very nearly inconsequential.

However, storing data in *non-archival* tiers (e.g., AWS S3, GCP Regional or Azure Hot tiers) can rapidly balloon costs. A genome stored in S3 for ten years costs $302.40, over 20x fold more than the archival tier. In order to examine how different archival strategies affect total cost, we created three different strategies (A, B, C in Table 1) and simulated the per-test cost across 20 years of operation, assuming a moderately high workload of 1,000 exomes per year (equivalent to 6TB of data per year). In strategy A, data is stored for 10 ​years ​at the target storage tier without any further archival. Strategy B and C simulate transition data from “hot” storage tiers to archival storage tiers at slow (2 years) or more aggressive (3 months) timeframe, respectively. These scenarios were input into ngscosts.info in order to generate a per-test marginal costs. As expected, more aggressive archival strategies resulted in more cost savings while preserving immediate access to recently generated files.

## 5 Discussion

Several key insights have emerged from the implementation of this tool and evaluation of different storage and archival strategies:

First, perhaps unsurprisingly, the cost to store data using cloud technologies is roughly linear with the amount of data stored. While growing clinical service, academic and research needs, and regulatory requirements may continually increase the total amount of data generated, laboratories should still pursue ways to reduce the total data footprint stored on the cloud. “Redundant” formats such as.fastq and.bam (if unmapped reads are included in.bam files) should be consolidated. In addition, formats for “lossy” compression (such as.cram [7]) may be employed to reduce file sizes of.bam files by 30–40%.

Second, cloud storage pricing varies dramatically (see Table 1). Of common options, the most expensive storage tier is nearly 21-fold more than the most economical option. We have found that prices between cloud vendors are largely competitive, although as of this writing, the Amazon Web Services’ Deep Glacier storage tier offers the lowest per-GB/month pricing.

Third, when evaluating data lifecycle policies, laboratories should prioritize moving data from immediately accessible “hot” tiers of storage to archival storage tiers. The cost to keep data in “hot” storage tiers (e.g., AWS S3, GCP Regional or Nearline, Azure “Hot” tiers) can rapidly become the majority of the marginal cost associated with storage, on account of the large price difference between tiers. For example, three months of AWS S3 storage is equal in price to nearly 6 years of AWS Deep Glacier storage. Similar effects (although less extreme) are seen with GCP and Azure cloud services as well.

To evaluate storage lifecycles in more depth, we describe three separate storage strategies evaluated using our tool in Table 1. In each scenario, the data generated is 1,000 exomes per year (equivalently, 6TB of data) with a target to store.bam files for at least 10 years. In Strategy “A” the data is stored directly in a single cloud storage tier for 10 years. This strategy is simple, and— depending on storage type selected— results in either the highest or the lowest 10-year cost per exome. However, low-cost strategy A variants are highly inflexible, as data is immediately archived and will not be accessible for near-term access or reanalysis if necessary. Strategies B and C propose the use of data lifecycle policies to transition data from standard storage tiers to archival storage tiers after two years or three months, respectively. These strategies may better accommodate laboratory workflows where access to recently generated data is needed, yet still provide substantial cost savings over 10 years.

Fourth, although often feared as “hidden cost” of cloud storage, retrieval costs are unlikely to be major contributors to the cost of cloud storage for most clinical labs. These costs are difficult to model, as re-access costs depend on the storage tier, the total amount of data re-accessed, the destination (e.g., within cloud or to local or other internet destinations), the speed or urgency at which data is requested (ranging from minutes to 48 ​h), and— for some archival type tiers— the amount of time data has been archived (i.e., cloud vendors may charge an “early re-access” fee for certain products). Nonetheless, in most cases where re-access occurs only rarely and without particular urgency, the cost to re-access data from archival tiers is typically less than 10% of the total cost. For example, for hypothetical 10% re-access rates using archival strategies B and C described above, the re-access costs as a percentage of each exome’s marginal cost ranges between approximately 1–5%. One exception to this general guideline should be noted for GCP’s Coldline storage platform, which has relatively high retrieval costs.

### 5.1 Limitations

Exact prediction of costs associated with storage of files on the cloud remains a challenging problem, which in large part depends on the specifics of the implemented solution and cloud vendor. Several specific limitations and assumptions are noted for this tool. Storage prices and other associated costs are fixed at current values, and the tool does not attempt to predict future price changes or changes to how storage costs are calculated. In addition, the tool assumes that expected file sizes (on a per-test basis) remain constant. Total costs and stored data are compounded monthly, while cloud storage vendors typically calculate costs using sub-hourly intervals. Tax, inflation and other overhead associated with cloud computing more generally are not specifically included.

In addition to per-gigabyte storage costs and re-access costs, Application Programming Interface (API) requests (e.g., to upload, retrieve or change storage tiers of a file) also contribute to the total cost of cloud storage. Genomic data storage typically involves large files (versus many small files), so the API request costs are typically a small fraction of the total cost of storage. Nonetheless, users of our tool should be aware that API request costs are not calculated.

Finally, in an effort to limit the configuration complexity, we have intentionally simplified some options. The number of cases re-accessed is split across all types of data, and early re-access costs (i.e., when data is accessed in cold storage tiers within a certain period of initial transition) are not modeled.

## 6 Conclusion

Cloud storage provides a scalable, technically advantageous and economical alternative to local storage of sequencing data for clinical genomics laboratories. Laboratory and informatics professionals can use https://ngstools.info to quickly evaluate marginal and total costs of cloud storage, and include difficult-to-calculate costs such as re-access, lifecycle management, and test volume growth. We have found that the primary drivers of cost are the total footprint of data as well as the storage tier and total time data remains in immediately accessible “hot” tiers of storage. In contrast, re-access costs of data do not substantially contribute to the total cost of data storage, although exceptions exist. In combination with unique technical advantages that cloud storage services provide (e.g., redundancy, auditing/access control, and encryption), laboratories should consider using cloud storage services for the sequencing and bioinformatics workflows.

## CRediT authorship contribution statement

**Niklas Krumm:** Conceptualization, Software, Writing - original draft. **Noah Hoffman:** Conceptualization, Writing - review & editing.

## Declaration of competing interest

The authors declare that they have no known competing financial interests or personal relationships that could have appeared to influence the work reported in this paper.
